# Chemical Compositions and In Vitro Antioxidant Activities of the Essential Oils of Sawdust and Resin-Rich Bark from Azorean *Cryptomeria japonica* (Cupressaceae)

**DOI:** 10.3390/antiox13060728

**Published:** 2024-06-15

**Authors:** Ana Lima, Filipe Arruda, Tanner Wortham, Alexandre Janeiro, Tânia Rodrigues, José Baptista, Elisabete Lima

**Affiliations:** 1 Institute of Agricultural and Environmental Research and Technology (IITAA), University of the Azores, 9700-042 Angra do Heroísmo, Portugal; ana.pr.lima@uac.pt (A.L.); filipe.mp.arruda@uac.pt (F.A.); alex-19961917@hotmail.com (A.J.); jose.ab.baptista@uac.pt (J.B.); 2 Department of Physics, Chemistry and Engineering (DCFQE), Faculty of Science and Technology, University of the Azores, 9500-321 Ponta Delgada, Portugal; 3 Department of Biology (DB), Faculty of Science and Technology, University of the Azores, 9500-321 Ponta Delgada, Portugal; tanyamsrod@gmail.com; 4 The Perfumery, 621 Park East Blvd, New Albany, IN 47150, USA; twortham@theperfumery.com

**Keywords:** Azores, *Cryptomeria japonica*, biomass residue valorization, sawdust, resin-rich bark, essential oil, monoterpene hydrocarbons, oxygenated sesquiterpenes, antioxidant properties, circular bioeconomy

## Abstract

In the Azores archipelago (Portugal), forest operations and wood industry generate large amounts of *Cryptomeria japonica* biomass residues (CJBR), which can be used to produce valuable essential oils (EOs). In this study, we evaluated the chemical composition and antioxidant activities of EOs from Azorean *C. japonica* sawdust (CJS) and resin-rich bark (CJRRB). The CJS and CJRRB EOs, obtained via hydrodistillation, showed different yield values (0.27% vs. 0.80% *v*/*w*, dry weight) and also different chemical profiles, as assessed using GC/MS. A total of 64 and 85 components were identified in CJS and CJRRB EOs, representing 95.7% and 96.9% of the total composition, respectively. The major components in CJS EO were oxygenated sesquiterpenes (mainly α+β-eudesmol, 1-epicubenol, and cubebol), while in CJRRB EO, the major components were monoterpene hydrocarbons, including α-pinene, δ-3-carene, and limonene (66.6% vs. 6.4% for oxygenated sesquiterpenes and 0% vs. 64% for monoterpene hydrocarbons, respectively). Antioxidant activity was estimated using (i) two radical-based assays, 2,2-diphenyl-1-picrylhydrazyl (DPPH) and 2,2′-azinobis-3-ethylbenzothiazoline-6-sulfonic acid (ABTS) radical scavenging activity, and (ii) a lipid model assay, β-carotene-linoleic acid bleaching activity (BCBA). Both CJS and CJRRB EOs exhibited concentration-dependent antioxidant activities, and their DPPH, ABTS, and BCBA EC_50_ values were 1107 vs. 1275 µg/mL, 260 vs. 498 µg/mL, and 1764 vs. 662 µg/mL, respectively. The results indicate that both EOs were able to exert antioxidant activity via different mechanisms of action. Therefore, Azorean CJS and CJRRB may be sustainable sources for antioxidant compounds. This study expands the chemical and biological knowledge of CJBR EOs and, consequently, adds more value to the *C. japonica* EO industry.

## 1. Introduction

Antioxidants play a crucial role in both the pharmaceutical and food industries as they are extensively used to prevent the onset and/or progression of a disease and food spoilage caused by reactive oxygen species (ROS), such as free radicals, hydrogen peroxide, and other peroxides [1]. However, there is growing global apprehension regarding the widespread consumption of synthetic antioxidants, such as butylated hydroxyl toluene (BHT) and butylated hydroxyl anisole (BHA), since it has been linked to various harmful effects on human health [2]. Therefore, nowadays, there is worldwide interest in looking for safe antioxidant products utilizing natural compounds from plants, such as essential oils (EOs), due to their generally recognized as safe (GRAS) status, wide acceptance by consumers, and their exploitation for multi-functional purposes across several industries [2,3,4]. In fact, EOs have been used therapeutically for centuries [5]. Moreover, in the last few decades, the antioxidant properties of EOs from several aromatic plants have been intensively investigated, evidencing that they may represent an effective eco-friendly alternative for medical, cosmetic, and/or food applications in the near future [2,3,4,6]. Such antioxidant abilities of EOs depend on their chemical composition, functional groups present in active components, and their synergistic interactions. Components with hydroxyl (particularly phenolic) groups or multiple bonds play a key role in the antioxidant properties of EOs [7]. Terpenes and their derivatives, i.e., terpenes containing different functional groups (also known as terpenoids) constitute the primary components found in EOs. Due to their chemical diversity, terpenes and terpenoids have a plethora of bioactivities and, thus, different physiological (e.g., hormones) and ecological (e.g., defense compounds) roles, as well as wide-spread industrial applications, ranging from flavors and fragrances to medicines [8,9]. Nevertheless, the composition and biological effects (including antioxidant properties) of an EO vary significantly among plants, even within the same species, owing to environmental and genetic variations. Furthermore, within a single plant, the chemical profile and biological properties of EOs can differ substantially based on the specific plant part. This diversity poses challenges in comprehensively understanding the effectiveness of EOs in a systematic manner [10,11].

On the other hand, using a single-substance/single-assay methodology to assess the antioxidant capacity of an EO produces relative results, with it being considered a reductive approach when dealing with complex plant extracts. Consequently, conducting multiple assays simultaneously with chemical characterization becomes imperative when evaluating EOs. This approach ensures a harmonious balance between sensory acceptability and functional properties [12]. For measuring free radical scavenging activity (FRSA), methods are usually grouped into two types, according to the chemical reaction involved: single electron transfer (SET) and hydrogen atom transfer (HAT). SET-based methods detect the ability of an antioxidant to transfer one electron to reduce a colored oxidant, including metals and radicals. Methods based on this principle include 2,2′-azinobis-3-ethylbenzothiazoline-6-sulfonic acid (ABTS) and 2,2-diphenyl-1-picrylhydrazyl (DPPH). Concerning the other approach, HAT-based methods, like β-carotene-linoleic acid bleaching activity (BCBA) assay, antioxidants and substrates compete for thermally generated peroxyl radicals. Furthermore, BCBA serves as a lipid peroxidation assay that mimics oxidative processes occurring in lipid-rich environments, such as biological membranes and food products containing fats and oils [13].

Nowadays, the increasing applications and markets for EOs could bring new opportunities for the sustainable management of unused forestry biomass residues (waste/by-products), such as the ones from *Cryptomeria japonica* (Thunb. ex L.f.) D. Don (Cupressaceae), known as a valuable, rich source of EOs [14]. This species, native to Japan, is a large evergreen, long-lived, monoecious conifer tree that was introduced into the Azores archipelago (Portugal) in the mid-19th century [15]. Curiously, it is noteworthy that the original forms of stumps and fallen logs of *C. japonica*, dating back centuries, remain nearly unchanged in Japanese forests due to the high resin concentration in the stems, providing robust resistance against fungal decay [16] and wood-feeding insects, in particular, termites [17]. Unlike other conifers, *C. japonica* does not have resin canals unless it has been injured. After injury, usually due to biotic factors, resin is exuded from the traumatized resin canal in the inner bark [18].

*Cryptomeria japonica* is, currently, the most important commercial forestry tree in the Azores, representing 60% of the total wood-producing forest area [19]. Therefore, the logging of Azorean *C. japonica* produces significant quantities of biomass residues (e.g., foliage, cones, and bark), which are often left unattended, representing an environmental problem. Furthermore, the timber industry, particularly sawmills, generates tons of biomass residues. Annually, 1.3 km^2^ of *C. japonica* cultivation area is approved for harvesting, yielding approximately 100,000 m^3^ of wood. It is estimated that around 30% of this wood becomes by-products, including sawdust and bark, without any or little commercial application [20]. However, these *C. japonica* biomass residues (CJBR) are still raw materials that can be converted into value-added products, such as EOs. In fact, the EOs extracted from different CJBR (mainly leaves, bark, and heartwood) exhibit several bioactivities, including molluscicide, mosquito larvicidal, mosquito repellent, termiticidal, antibacterial, antifungal, and neuropharmacological effects, namely anxiolytic, analgesic, and soothing properties [21,22,23,24,25,26,27,28].

Nevertheless, *C. japonica* foliage is by far the plant organ most studied and used by Azorean wood producers to obtain EOs, with several applications, such as in the fragrance industry and aromatherapy. As part of our continuing strategy of the valorization of Azorean CJBR, thus contributing to increasing the local circular economy, we recently reported that EOs obtained via hydrodistillation (HD) processes from various aerial plant parts (leaves, foliage, female cones, and male cones) exhibit multi-bioactivities, such as natural biocides [29] and antioxidants [19]. However, studies on the chemical composition and biological activities of EOs of sawdust and bark from *C. japonica* remain limited. In this context, and knowing that the biological activity of EOs depends mainly on their chemical composition, which, as reported previously, is influenced by the plant part, the purposes of the present study were to compare the chemical composition of the EOs extracted via HD from Azorean *C. japonica* sawdust (CJS) and resin-rich bark (CJRRB) and compare their in vitro antioxidant activities, evaluated via FRSA (DPPH and ABTS) and BCBA assays.

## 2. Materials and Methods

### 2.1. Chemicals and Reagents

Anhydrous sodium sulfate (Na_2_SO_4_), 2,2′-azinobis-3-ethylbenzothiazoline-6-sulfonic acid (ABTS), 2,2-diphenyl-1-picrylhydrazyl (DPPH), gallic acid, β-carotene, linoleic acid, and Tween 20 were purchased from Sigma–Aldrich (St. Louis, MO, USA). Methanol (≥99.8%), ethanol (96%), and chloroform (≥99%) were purchased from Riedel-de Häen (Aktiengesellschaft, Seelze, Germany).

### 2.2. Sample Collection and Essential Oil Isolation

**Figure 1:**
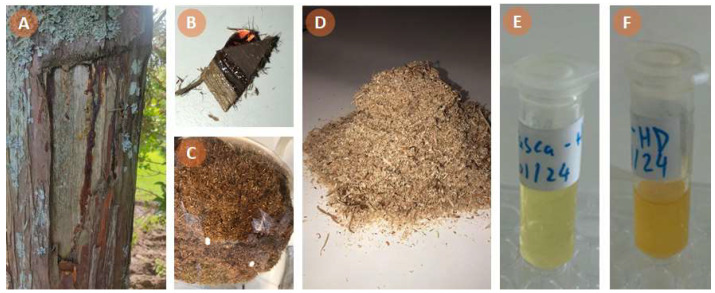
Azorean *Cryptomeria japonica* and samples: (**A**) tree exhibiting bark damage; (**B**) a piece of resin-rich bark; (**C**) resin-rich ground bark sample; (**D**) sawdust sample; (**E**) essential oil from the resin-rich bark sample; (**F**) essential oil from the sawdust sample.

The CJRRB sample was collected from a wound on a *C. japonica* tree (Figure 1A,B), standing at approximately 7 m in height and with a breast height diameter of 23 cm, in November 2023. This tree was located in Lomba da Maia, at latitude 37°49′07.7″ N, longitude 25°21′33.2″ W, and an altitude of 330 m, on São Miguel Island, Azores archipelago, Portugal. The climate in this archipelago is characterized by mild temperatures, high relative humidity, regular rainfall, and strong winds, with an average temperature of 13 °C in winter and 24 °C in summer [30]. The CJRRB sample was immediately brought to the laboratory at the University of the Azores, cleaned of lichens, and then shade-dried at room temperature (20 °C) in a well ventilated area. Following drying, the CJRRB sample was pulverized into powder (Figure 1C) using a mechanical grinder.

The CJS sample, i.e., the woodmeal of *C. japonica* (Figure 1D), was obtained from the local carpentry industry on São Miguel Island. The sample was air-dried at room temperature (20 °C) prior to EO extraction.

The EOs from CJS and CJRRB (Figure 1E,F) were extracted using HD in a modified Clevenger-type apparatus, in accordance with the European Pharmacopoeia method [31]. In detail, the sample-to-water ratio was 1:10 g/mL, and the distillation time was approximately 3 h, starting with the first droplet of distillate. Each HD process was performed in triplicate. After drying over anhydrous Na_2_SO_4_, the EOs were stored at 4 °C in the dark before analysis. The EO yield (%, *v*/*w*) was calculated on a dry weight (d.w.) basis.

### 2.3. Essential Oil Composition Analysis

The chemical composition of the EO samples was determined using gas chromatography/mass spectrometry (GC/MS) on a Shimadzu GCMS–QP2010 Ultra gas chromatograph/mass spectrometer, equipped with a ZB–5MSPlus (5% phenyl; 95% methyl siloxane) capillary column (60 m × 0.25 mm i.d.; film thickness of 0.25 µm) from Phenomenex Inc. (Torrance, CA, USA). The oven’s temperature was increased from 50 °C to 260 °C at a rate of 2 °C/min and was then held for 5 min at the final temperature. The injector and detector temperatures were set at 260 °C, and the transfer line temperature was set at 300 °C. A volume of 0.1 μL of EO sample dissolved in methylene chloride (0.1 g/mL) was injected for analysis at a split ratio of 24.4:1. Helium was used as the carrier gas at a linear velocity of 36.3 cm/s. The mass spectra were recorded over the 40–400 atomic mass units (amu) range at 0.3 scans per second, with an ionization energy of 70 eV and the ion source temperature at 260 °C [19]. The identification of the EO components was assigned by matching (i) their recorded mass spectra with the standard mass spectra from several libraries (a lab-made library and FFNSC4.0, NIST11, and Wiley10 libraries) and (ii) their retention indices (RI), calculated according to ISO 7609 [32], relative to a homologous series of *n*-alkanes (C_7_–C_33_). The relative concentration of each EO component was quantified by integrating total ion current (TIC) chromatogram peaks without correction factors as the mean values of three injections from each EO sample.

### 2.4. In Vitro Antioxidant Activity

The antioxidant activity of the EOs was determined by using DPPH, ABTS, and BCBA assays. All data collected for each assay are the averages of three determinations of three independent experiments.

#### 2.4.1. DPPH Free Radical-Scavenging Activity (FRSA) Assay

The FRSA of the EOs, at different concentrations (0.024–25 mg/mL), and gallic acid (positive control) was determined following the procedure reported by Chen et al. [33], with slight modifications [19]. In brief, 0.1 mL of each EO or gallic acid was allowed to react with 0.1 mL of DPPH solution (0.08 mg/mL in methanol) in the well of a 96-well plate. The reaction mixture was shaken vigorously and left to stand at room temperature in the dark. After 30 min, the absorbance (Abs) was measured at 520 nm in a microplate reader (Thermo Fisher Scientific, Waltham, MA, USA), against a blank containing all reagents except for the test samples. The FRSA was calculated as a percentage of DPPH discoloration using the following Equation (1):

(1) $$FRSA\left(\%\right)=1-\left(\frac{{Abs}_{sample}}{{Abs}_{blank}}\right)\times 100$$

The results are expressed as EC_50_ values (µg/mL), which is defined as the sample concentration needed to quench 50% of the DPPH stable free radicals. A lower EC_50_ value is indicative of higher antioxidant activity.

#### 2.4.2. ABTS Free Radical-Scavenging Activity (FRSA)

The FRSA of the EOs, at different concentrations (0.024–25 mg/mL), and gallic acid (positive control) was also determined by measuring their ability to quench the ABTS radical cation, according to the method reported by Re et al. [34], with slight modifications. Briefly, a reaction of 7.0 mM ABTS and 2.45 mM K_2_S_2_O_8_, kept in the dark for 16 h at room temperature, was used to obtain ABTS radicals. Afterward, the ABTS solution was diluted with methanol until the Abs reached 0.7 at 734 nm. Then, a 0.1 mL aliquot of each EO or gallic acid was added to 0.1 mL of ABTS solution. The plate was shaken and incubated in the dark for 6 min at room temperature, and then Abs was measured at 734 nm in a microplate reader (Thermo scientific Multiskan FC, Thermo Fisher Scientific), against a blank containing all reagents except for the test samples. Scavenging capacity was calculated using Equation (1) described above, and the results are expressed as EC_50_ values (µg/mL).

#### 2.4.3. β-Carotene-Linoleic Acid Bleaching Activity (BCBA) Assay

The BCBA of the EOs, at different concentrations (0.03–8.33 mg/mL), and gallic acid (positive control) was assessed following the method reported by Miller [35], with minor adjustments that were necessary for the introduction of a microtiter plate for higher throughput [7]. In a boiling flask, 25 µL of β-carotene solution (20 mg/mL in chloroform) was mixed with 20 µL of linoleic acid, 200 mg of Tween 20, and 500 µL of chloroform. The chloroform was evaporated for 60 min using a rotary evaporator at 40 °C. Subsequently, 25 mL of distilled water was slowly added to the flask with vigorous stirring to form an emulsion. Emulsion aliquots (250 µL) were mixed with 50 µL of each EO or gallic acid. The mixture was incubated for 3 h at 50 °C, during which Abs was measured at 450 nm, before (t = 0) and after incubation, against a blank that consisted of an emulsion without β-carotene. The control samples contained 50 μL of water instead. The BCBA was calculated as percent inhibition relative to the control using the following Equation (2):

(2) $$BCBA\left(\%\right)=\left[\frac{\left(S_{t}-C_{t}\right)}{C_{0}-C_{t}}\right]\times 100$$

where S_t_ and C_t_ are the Abs of the sample and the control after 3 h of incubation, respectively, and C_0_ is the control Abs measured at zero minutes (t = 0). The kinetics of this activity allowed us to determine the sample’s concentration corresponding to 50% inhibition of β-carotene discoloration (EC_50_ value).

### 2.5. Statistical Analysis

The data are expressed as the mean ± standard deviation (SD). The normal distribution of continuous variables was tested with a Shapiro–Wilk test. Analysis of variance was performed using the ANOVA procedure, and Duncan’s new multiple-range test was used to compare the EOs’ antioxidant capacities determined using the ABTS, DPPH, and BCBA assays. Additionally, Pearson’s linear coefficient of correlation was calculated in order to characterize the relationship between antioxidant capacities detected using different assays. The level of statistical significance was set at *p* < 0.05 for two-sided testing. All analyses were conducted using IBM SPSS Statistics version 27.0 software (SPSS Inc., Chicago, IL, USA).

## 3. Results and Discussion

### 3.1. Essential Old Yield and Chemical Composition

The yields of CJS and CJRRB EOs were 0.27% and 0.80% (*v*/*w*, d.w.), respectively. Similar results have already been reported by Cheng et al. [23] for *C. japonica* from Taiwan. Lower yield values are usually reported for *C. japonica* bark (CJB) EO [36], but it is noteworthy that the bark sample in this study is atypical (very rich in resin) and originated from a wound on the *C. japonica* tree. The CJS EO was much more viscous and had a darker color compared to the CJRRB EO, which appeared pale yellow (Figure 1E,F). Both EOs possessed pleasant odors albeit distinct.

**Figure 2:**
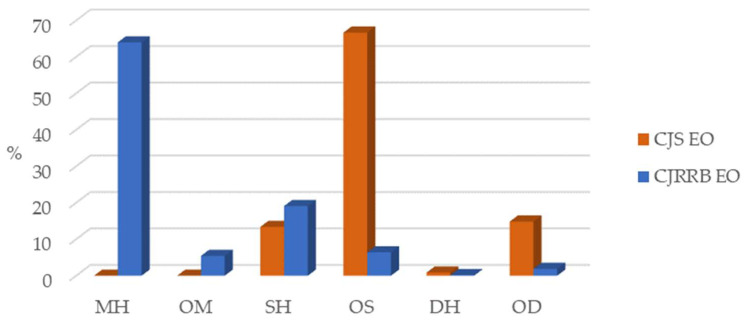
Grouped components (%) of the essential oils (EOs) isolated via the hydrodistillation of Azorean *Cryptomeria japonica* sawdust (CJS) and resin-rich bark (CJRRB). Legend: MH—monoterpene hydrocarbons; OM—oxygenated monoterpenes; SH—sesquiterpene hydrocarbons; OS–oxygenated sesquiterpenes; DH—diterpene hydrocarbons; OD–—oxygenated diterpenes.

The results of the GC/MS analyses of CJS and CJRRB EOs are listed in Table 1. In total, 64 and 85 components were identified in these EOs, representing 95.7% and 96.9% of the total detected constituents, respectively. Figure 2 shows the percentage of CJS and CJRRB EO components grouped according to their chemical class.

As shown in Table 1 and Figure 2, the CJS EO was mainly characterized by oxygenated sesquiterpenes (OS), followed by oxygenated diterpenes (OD), sesquiterpene hydrocarbons (SH), and diterpene hydrocarbons (DH) (66.64%, 14.83%, 13.38%, and 0.86%, respectively). Neither monoterpene hydrocarbons (MH) nor oxygenated monoterpenes (OM) were identified in the CJS EO. The major components (>5.0%) in the CJS EO were α+β-eudesmol (13.5%), 1-epicubenol (10.7%), cubebol (6.8%), δ-cadinene (6.4%), τ-cadinol (5.9%), and sandaracopimarinol (5.5%). Similar results were reported by Narita et al. [38] for Japanese stumps of *C. japonica* and by Ho et al. [39]. Additionally, findings related to those of *C. japonica* heartwood from Faial Island (Azores) were reported by Moiteiro et al. [36]. On the other hand, different results were also reported by Cheng et al. [23], where the main terpene class of *C. japonica* heartwood EO was SH, mainly a δ-cadinene compound. These observed differences may be related to genetic and environmental factors (e.g., geographical location and season). It is also worth noting that the studied CJS sample (provided by the local carpentry industry) may contain a mixture of sapwood and heartwood, and possibly traces of bark, which also makes comparisons with other studies more difficult.

Phytochemical analysis of the CJRRB EO revealed that this EO was dominated by MH (63.97%), mainly due to its α-pinene content (42.7%), followed by limonene (8.9%) and δ-3-carene (6.0%). Similar results were reported by Yatagai et al. [18] in Japanese CJB. On the other hand, different results were also documented in the studies of Moiteiro et al. [36] and Ho et al. [39], where the latter identified camphor as the major component in the CJB EO. As previously mentioned, it is worth noting that the bark sample used in this study is atypical, being exceptionally rich in resin, and it originated from a wound on the *C. japonica* tree, which could explain the high volatile compound content. Concerning the other terpene groups, SH was the second most representative group (19.09%) in the CJRRB EO, followed by OS (6.39%), OM (5.42%), OD (1.89%), and DH (0.16%).

Although the percentage of SH was comparable in both CJS and CJRRB EOs (13.4% vs. 19.1%), it is worth noting that the content of DH was five times higher in the CJS EO than in the CJRRB EO (0.87% vs. 0.16%). Additionally, the content of the OD was approximately eight times higher in the CJS EO than in the CJRRB EO (14.8% vs. 1.9%).

### 3.2. Essential Oils’ Antioxidant Activities

The antioxidant activities of the studied EOs using DPPH and ABTS radical scavenging assays, as well as the BCBA assay, are shown, as EC_50_ values, in Table 2 and compared to gallic acid as a positive control.

Both EOs exhibited weak activity for DPPH and ABTS radical scavenging activity compared with the standard antioxidants. However, the CJS EO presented higher FRSA activity than the CJRRB EO in the ABTS assay, aligning with the findings of Ho et al. [39]. As stated by these authors, this activity may be attributed to the ferruginol content (a phenolic diterpene), which, in our study, was found to be highest in the CJS EO (3.6% vs. 0.9%). In fact, it has already been reported that diterpenes, with phenolic groups, show higher FRSA effects than MH [12,40]. However, no difference in antioxidant activity between the studied EOs was observed in the DPPH assay. Although the ABTS method is similar to the DPPH assay, antioxidant levels determined using the ABTS method were significantly lower than those reported in the DPPH assay. Similar differences in the antioxidant activity of EOs, in both of these methods, have been previously reported [41]. Overall, in this study, it seems that the ABTS method is more reliable than the DPPH method. In fact, the ABTS radical is reactive toward most antioxidants, and it is soluble in both aqueous and organic solvents, with it being a useful tool in determining the antioxidant activity of both hydrophilic and hydrophobic antioxidants, while DPPH dissolves only in polar matrices [42], and *C. japonica* EOs are chiefly hydrophobic due to their high MH content. Moreover, when assessing the SET-based methods, the ABTS method emerges as the preferred technique for determination compared to the DPPH method. This preference stems from its stronger correlation with the donors of both protons and electrons (such as OS and OD in CJS EO) necessary for neutralizing these radicals. Nevertheless, both FRSA methods exhibited a linear correlation in this study (r = 0.829, *p* < 0.001), as is usual.

However, when compared with the other plant parts of *C. japonica*, namely immature female cones (IFC), both EO samples in this study exhibited less scavenging activity in the DPPH assay, with EC_50_ values of 1.1–1.3 mg/mL vs. 0.67 ± 0.24 mg/mL for IFC. These results may be associated with the content of γ-eudesmol and nezukol, which are both higher in the IFC samples [19].

As already reported [12], the results of a single assay give only a reductive suggestion of the antioxidant properties of an EO. Therefore, BCBA assay was also performed in both samples, as a closer model to the real lipid system occurring in food products and in human cells. However, in the BCBA assay, different results emerged, i.e., the CJS EO exhibited lower antioxidant activity compared to the CJRRB EO. This is not the first study where it has been observed that EOs with high MH content were more effective in BCBA assay than in DPPH assay, maybe as a consequence of a higher specificity of the assay for lipophilic compounds [12]. Furthermore, these results are not strange if we consider that the antioxidant activity in BCBA assay is determined by a different mechanism, i.e., two competitive chemical reactions in which the examined antioxidant(s) or β-carotene take part. In fact, MH and OM, such as α-pinene, myrcene, *p*-cymene, β-phellandrene, limonene, δ-3-carene, linalool, *trans*-pinocarveol, borneol, and α-terpineol, are more likely to possess C=C double bonds or π-conjugated molecules (similar to β-carotene), which is associated with the loss of the allylic hydrogen atom. Consequently, they are also able to form radical adducts with peroxyl radicals and exhibit antioxidant properties [41]. Although α-pinene is known as a potent antioxidant agent [43], it has been observed that the antioxidant properties of an EO do not always depend on the properties of its main components since this activity can be modulated by other components (through synergy, additivity, and/or antagonism mechanisms) of the EO [41].

Overall, in this study, when compared to the standard antioxidant (gallic acid), both EOs exhibited stronger antioxidant activity in the BCBA assay than in the FRSA assays. Similar results have also been documented for other plant parts of Azorean *C. japonica*, where α-pinene is typically the main compound [19].

**Figure 3:**
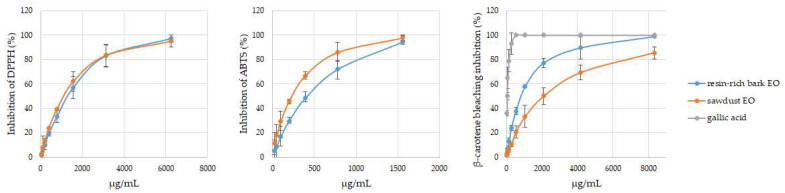
Antioxidant activity of the essential oils (EOs) isolated via the hydrodistillation of Azorean *Cryptomeria japonica* sawdust and resin-rich bark, at different concentrations, measured using the DPPH, ABTS, and BCBA methods. There are no statistically significant differences when the curves and standard deviations overlap.

As expected, the ABTS method did not correlate with the BCBA assay (r = 0.363; *p* = 0.336), whereas the DPPH method did correlate with it (r = 0.783; *p* = 0.012). Once again, the ABTS method seems more reliable than the DPPH method. Similar findings have been previously reported [42], indicating variations in antioxidant capacity when assessed using different in vitro assays. Nevertheless, both studied EOs exhibited antioxidant activities in a concentration-dependent manner, in all assays, as shown in Figure 3.

In sum, Azorean CJS EO contains a significant level of important compounds, such as 1-epicubenol and δ-cadinene, which possess several biological properties that affect human health and wood durability [44,45]. This EO was the richest in compounds with hydroxyl groups (such as OS and OD), which best explains the observed FRSA. On the other hand, the chemical composition of CJRRB EO reveals a high concentration of MH and OM, such as α-pinene, δ-3-carene, and limonene, which have already been associated with antimicrobial, repellent, and insecticidal properties [27,46]. Both EOs displayed distinct antioxidant properties, which are linked to their different chemical compositions. Specifically, the CJRRB EO demonstrated superior activity in the lipid BCBA assay. This indicates its capacity to inhibit the oxidation of unsaturated fatty acids, rendering it a potential application within the food industry. However, both EOs can have applications in the food, cosmetic, and medical industries.

The present study, however, has some limitations. First, the studied CJRRB sample may not be representative of the Azorean CJB, whereas the CJS sample (supplied by the local carpentry industry) may include a mixture of sapwood and heartwood and even traces of bark. Second, antioxidant assays are in vitro models and do not assess all of the antioxidant activities in food/organisms. Lastly, given the scarcity of studies with timber waste samples from Azorean *C. japonica*, as well as potential variations in the composition of their EOs due to environmental factors, further research will be necessary to verify the reproducibility of these results.

## 4. Conclusions

The *C. japonica* timber industry, particularly sawmills, produces tons of biomass residues annually, including sawdust and bark, without any or little commercial application. Thus, repurposing these residues to create value-added products, including EOs, is imperative. To the best of our knowledge, this is the first study that reports the phytochemistry and antioxidant activity of EOs from *C. japonica* sawdust and resin-rich bark obtained from São Miguel Island, Azores.

In the phytochemical analysis of the sawdust EO sample, a noteworthy revelation is the prevalence of OS, namely α+β-eudesmol, 1-epicubenol, and cubebol, which emerged as the principal compounds.

In contrast, the investigation into the phytochemical makeup of resin-rich bark EO samples disclosed the presence of distinct compounds, namely MH and OM. Notably, α-pinene stood out as the predominant constituent, accounting for a significant proportion of 43% within the EO.

Overall, both EOs were characterized by the presence of numerous bioactive compounds, which, in turn, could have various applications in health, food, and pest control. Moreover, in this study, both EOs were able to exert antioxidant activity via different mechanisms of action, as revealed by the different applied tests. Thus, the results indicate that both EOs, if demonstrated as safe, could be alternative raw materials for the food industry and used as medicinal products for pharmaceutical applications.

Hence, it appears reasonable to transform timber industry residues into environmentally friendly EOs, thereby enhancing the local sustainable circular economy. This approach augments timber product diversity and efficiency, minimizes waste, and mitigates environmental impact.

## Figures and Tables

**Table 1:** Composition of the essential oils (EOs) isolated via the hydrodistillation of Azorean *Cryptomeria japonica* sawdust (CJS) and resin-rich bark (CJRRB).

No.	Class and Component	RT	RI_L_	RI_C_	Relative Content (%)	
					CJS EO	CJRRB EO
	Monoterpene hydrocarbons					
1	Tricyclene	11.92	921	916		0.14
2	α-Thujene	12.07	924	919		0.02
**3**	**α-Pinene**	12.54	932	927		**42.74**
4	α-Fenchene	13.29	945	941		0.40
5	Camphene	13.38	946	942		0.49
6	Thuja-2,4(10)-diene	13.58	953	946		0.05
7	*m*-Cymene	14.57		963		0.09
8	β-Pinene	14.96	974	970		1.80
9	Myrcene	15.59	988	981		2.52
**10**	**δ-3-Carene**	16.82	1008	1003		**6.02**
11	α-Terpinene	17.32	1014	1010		0.03
12	*o*-Cymene	17.48	1022	1013		0.02
13	*p*-Cymene	17.80	1020	1018		0.17
**14**	**Limonene**	18.11	1024	1022		**8.93**
15	β-Phellandrene	18.21	1025	1024		0.21
16	γ-Terpinene	19.97	1054	1050		0.03
17	Isoterpinolene	21.52	1085	1074		0.02
18	Terpinolene	21.79	1086	1078		0.17
19	*p*-Cymenene	22.10	1089	1082		0.12
	Oxygenated monoterpenes					
20	Fenchone	22.00	1083	1081		0.03
21	Linalool	22.73	1095	1092		0.10
22	endo-Fenchol	24.06	1114	1111		0.03
23	α-Campholenal	24.59	1122	1119		0.17
24	*cis*-Limonene oxide	24.99	1132	1125		0.12
25	*trans*-Pinocarveol	25.55	1135	1133		0.15
26	Camphor	25.98	1141	1139		0.21
27	Camphene hydrate	26.53	1145	1147		0.04
28	Pinocamphone	26.91	1158	1152		0.05
29	Borneol	27.68	1165	1163		0.29
30	Isopinocamphone	27.98	1172	1168		0.12
31	Terpinen-4-ol	28.30	1174	1172		0.35
32	*p*-Cymen-8-ol	28.72	1179	1178		0.05
33	α-Terpineol	29.30	1186	1187		0.87
34	Verbenone	30.10	1204	1198		0.02
35	*trans*-Carveol	30.93	1215	1210		0.06
36	Thymol methyl ether	32.28	1232	1230		0.07
37	Carvone	32.64	1239	1235		0.03
38	Linalyl acetate	33.03	1254	1241		0.10
39	Piperitone	33.30	1249	1245		0.02
40	Bornyl acetate	35.38	1287	1275		0.67
41	Isobornyl acetate	35.54		1277		0.03
42	Methyl myrtenate	36.11	1293	1286		0.52
43	Thujyl acetate	36.73	1295	1295		0.03
44	α-Terpinyl acetate	39.52	1346	1337		1.29
	Sesquiterpene hydrocarbons					
45	α-Cubebene	39.75	1345	1340	0.19	0.04
46	α-Copaene	41.44	1374	1366		0.31
47	β-Cubebene	42.23	1387	1378		0.19
48	(*Z*)-β-Caryophyllene	43.04	1408	1390		0.04
49	Longifolene	43.60	1407	1399		0.69
50	*cis*-Muurola-4(14),5-diene	44.15	1465	1408		0.08
51	(*E*)-β-Caryophyllene	44.24	1417	1409	0.03	0.14
52	β-Copaene	44.89	1430	1419	0.09	0.19
53	α-Guaiene	45.23	1437	1425		0.02
54	*trans*-Murrola-3,5-diene	46.15	1451	1438	0.59	0.87
55	α-Humulene	46.55	1452	1445	0.04	0.16
56	10-beta-H-Cadina-1(6),4-diene	47.59		1461	1.33	1.52
57	*trans*-Cadina-1(6),4-diene	47.77	1475	1464	0.09	0.24
58	*trans*-Muurola-4(14),5-diene	48.81	1493	1481	1.14	1.22
59	α-Muurolene	49.23	1500	1488	0.54	2.58
60	β-Bisabolene	49.81	1505	1497	0.02	0.08
61	γ-Cadinene	50.04	1513	1502		0.18
**62**	**δ-Cadinene**	50.44	1522	1507	**6.42**	**7.23**
63	*trans*-Calamenene	50.55	1521	1510		1.16
64	*cis*-Calamenene	50.63	1528	1511	0.48	
65	Zonarene	50.71	1528	1511	1.24	1.06
66	*trans*-Cadina-1,4-diene	51.27	1533	1521	0.71	0.75
67	α-Calacorene	51.77	1544	1523	0.21	0.10
68	α-Calacorene isomer	51.38		1524	0.06	
69	β-Calacorene	53.00	1564	1529	0.09	0.20
70	Cadalene	59.35	1675	1660	0.11	0.04
	Oxygenated sesquiterpenes					
71	epi-Cubebol	49.06	1493	1486	4.73	0.79
**72**	**Cubebol**	50.26	1514	1504	**6.76**	**1.28**
73	Elemol	52.19	1548	1536	2.59	0.03
74	(*E*)-Nerolidol	52.87	1561	1549		0.62
75	Spathulenol	53.87	1577	1566	0.05	
76	Caryophyllene oxide	54.18	1582	1570	0.08	0.27
77	Gleenol	54.48	1586	1575	1.52	0.34
78	*cis*-Muurol-5-en-4-α-ol	54.56	1559	1578	0.31	
79	*trans*-Muurol-5-en-4-α-ol	54.67		1579	0.54	
80	Humulene epoxide II	55.80	1608	1597	0.10	0.19
81	Eudesm-5-en-11-ol	55.99		1602	0.02	
82	1,10-di-Epicubenol	56.12	1618	1604	0.07	
83	10-epi-γ-Eudesmol	56.52	1622	1611	0.05	
**84**	**1-Epicubenol**	56.87	1627	1615	**10.74**	**1.93**
85	Agarospirol	57.02	1646	1620	0.66	
86	γ-Eudesmol	57.08	1630	1621	2.22	
87	Hinesol	57.52	1640	1629	0.10	
**88**	**τ-Cadinol**	57.71		1632	**5.90**	
89	epi-α-Cadinol	57.75	1638	1633	2.28	
90	δ-Cadinol	57.93		1636	4.32	0.40
**91**	**β+α-Eudesmol**	58.40	1649/1652	1643	**13.54**	**0.54**
92	Selin-11-en-4-α-ol	58.58	1658	1647	2.74	
93	7-epi-α-Eudesmol	58.73	1662	1650	0.03	
94	Campherenone	59.58		1665	0.10	
95	Amorpha-4,9-dien-2-ol	60.69		1685	0.39	
96	Juniper camphor	60.74		1686	0.35	
97	5-Hydroxy-*cis*-Calamenene	61.70	1713	1703	0.05	
98	β-Bisabolenal	62.29	1768	1714	4.03	
99	(6*S*)-2,10-Bisaboladien-1-one	63.16		1730	0.05	
100	Aristol-9-en-8-one	65.04		1765	0.04	
101	α-Eudesmol acetate	65.32	1794	1770	0.07	
102	2,7(14),10-Bisabolatrien-1-ol-4-one	68.57	1844	1832	0.08	
103	11-acetoxy-Eudesman-4-α-ol isomer	71.40		1888	0.03	
104	2,7(14),10-Bisabolatrien-1-ol-4-one isomer	71.92		1898	1.95	
105	11-acetoxy-Eudesman-4-α-ol	73.11		1922	0.15	
	Diterpene hydrocarbons					
106	Sandaracopimara-8(14),15-diene	74.48	1968	1950	0.17	
107	Phyllocladene	77.20	2016	2005	0.04	0.03
108	Kaur-16-ene	78.30	2042	2030	0.03	
109	Abitatriene	78.65	2055	2036	0.13	0.13
110	Abitadiene	80.16	2087	2070	0.49	
	Oxygenated diterpenes					
111	Manool oxide	75.78	1987	1977		0.02
112	Sandaracopimarinal	84.53	2184	2164	3.03	0.09
113	Phyllocladanol	85.82	2209	2193	1.68	0.26
114	Sandaracopimarinol isomer	87.46		2233	0.05	
**115**	**Sandaracopimarinol**	88.30	2269	2253	**5.48**	
116	6,7-Dehydroferruginol	90.21	2315	2298	0.93	0.62
117	*trans*-Ferruginol	90.37	2331	2301	3.64	0.90
118	*trans*-Ferruginol acetate	91.26	2363	2323	0.02	
	**Identified components (%)**				95.71	96.92

Standard error (SE) < 0.7% for compounds with percentage < 30%. For compounds > 30%, SE < 2%. Components higher than 5.00% are highlighted in boldface. Legend: RI_L_—retention indices from the literature [37]; RI_C_—retention indices on a ZB–5MSPlus capillary column; RT—retention time (minutes) values on the same column.

**Table 2:** Antioxidant activity of the essential oils (EOs) isolated via the hydrodistillation of Azorean *Cryptomeria japonica* sawdust and resin-rich bark.

Samples	EC_50_, µg/mL		
	DPPH	ABTS	BCBA
Sawdust EO	1107 ± 94 ^b^	261 ± 6 ^b^	1764 ± 388 ^c^
Resin-rich bark EO	1275 ± 347 ^b^	498 ± 20 ^c^	662 ± 37 ^b^
Gallic acid	1.93 ± 0.09 ^a^	1.13 ± 0.01 ^a^	38 ± 5 ^a^

Values are the mean ± SD (*n* = 3). Different superscript letters in the same column indicate statistically significant differences at *p* < 0.05. Legend: DPPH—2,2-diphenyl-1-picrylhydrazyl; ABTS—2,2′-azinobis-3-ethylbenzothiazoline-6-sulfonic acid; BCBA—β-carotene-linoleic acid bleaching activity.

## Data Availability

Data are contained within the article.

## Abbreviations

Abs, absorbance; ABTS, 2,2′-azinobis-3-ethylbenzothiazoline-6-sulfonic acid; BCBA, β-carotene-linoleic acid bleaching activity; CJB, *Cryptomeria japonica* bark; CJBR, *Cryptomeria japonica* biomass residues; CJRRB, *Cryptomeria japonica* resin-rich bark; CJS, *Cryptomeria japonica* sawdust; DH, diterpene hydrocarbons; DPPH, 2,2-diphenyl-1-picrylhydrazyl; d.w., dry weight; EO, essential oil; FRSA, free radical-scavenging activity; GC/MS, gas chromatography/mass spectroscopy; HAT, hydrogen transfer atom; HD, hydrodistillation; MH, monoterpene hydrocarbons; OD, oxygenated diterpenes; OM, oxygenated monoterpenes; OS, oxygenated sesquiterpenes; RI, retention indices; SET, single electron transfer; SH, sesquiterpene hydrocarbons.
